# Blast crisis Ph+ chronic myeloid leukemia with *NUP98/HOXA13* up-regulating *MSI2*

**DOI:** 10.1186/1755-8166-7-42

**Published:** 2014-06-20

**Authors:** Danika Di Giacomo, Valentina Pierini, Gianluca Barba, Veronica Ceccarelli, Alba Vecchini, Cristina Mecucci

**Affiliations:** 1 Hematology and Bone Marrow Transplantation Unit, University of Perugia, Polo Unico S. Maria della Misericordia, Perugia, Italy; 2 Department of Internal Medicine, University of Perugia, Perugia, Italy

**Keywords:** Blast crisis CML, Fusion transcript, *MSI2*, *HOXA* genes

## Abstract

**Background:**

Musashi2(Msi2)-Numb pathway de-regulation is a molecular mechanism underlying the transition of chronic phase Ph + CML to deadly blast crisis, particularly in cases with a *NUP98/HOXA9* fusion from a t(7;11)(p15;p15). This study provides new insights on the mechanisms cooperating in driving *MSI2* over-expression and progression of Ph-positive CML.

**Results:**

Herein we describe a t(7;11)(p15;p15) originating a *NUP98* fusion with *HOXA13,* at 7p15, in a 39 year-old man in blast crisis of Ph-positive CML. Both *MSI2* and *HOXA9* were evaluated by quantitative RT-PCR in our patient and in a series of haematological malignancies. Up-regulation of both genes emerged only in the presence of *NUP98/HOXA13* gene fusion. However, over-expression of *MSI2*, but not *HOXA9*, was found in 2 cases of Ph + blast crisis with additional chromosome aberrations other than t(7;11). To determine the mechanisms underlying *MSI2* over-expression in our patient we performed Chromatin Immunoprecipitation and found that NUP98/HOXA13 fusion protein deregulates *MSI2* gene by binding its promoter.

**Conclusions:**

To the best of our knowledge, this is the first molecular characterization of *NUP98/HOXA13* fusion in blast crisis of Ph + CML. Our findings suggest cooperative mechanisms of *MSI2* over-expression driven by HOXA proteins and strongly supports *MSI2* as a prognostic marker and a candidate in target treatment of CML.

## Background

*MSI2* gene (17q22) is a member of the *Musashi (Msi)* family, which is well conserved during mammals evolution and distributed in the stem cell compartment of neural [1], hematopoietic [2], pancreatic [3] and epithelial tissues [4,5]. Two translocations involving *MSI2* gene at 17q22 have been described in myeloid malignancies [6,7] and more than 50 point mutations have been reported in solid tumors and cell lines (COSMIC database [8], cBioPortal for Cancer Genomics [9]) [10]. *Msi2* expression is activated by cooperation between HoxA9 and Meis1 in *Meis1*-immortalized hematopoietic progenitors [11]. In chronic myeloid leukemia (CML) Ito et al. [12] showed the *Nup98/HoxA9* fusion contributed to blast crisis through HoxA9 homeodomain binding to the *Msi2* promoter resulting in gene over-expression, down-regulation of the Notch1 inhibitor Numb, and loss of the proliferation/differentiation equilibrium in hematopoietic stem cells (HSCs) [13]. De-regulation of the Musashi-Numb-Notch1 signaling axis is associated with poor prognosis in CML [12], acute myeloid leukemia (AML) [14] and B-cell acute lymphoblastic leukemia (B-ALL) [15]. In addition to *HOXA9*, two genes of the *HOXA* cluster at 7p15, i.e., *HOXA11*[16] and *HOXA13*[17], rearrange with *NUP98* in leukemia. All fusion transcripts contain the NUP98 N-terminal, with the FG/GLFG domains, that mediate both RNA and protein transport, and the HOX homeodomain, with its DNA binding ability [18]. Thus all these chimeric proteins exhibit their oncogenic potential via transcriptional activation of downstream genes [19]. Remarkably a single t(7;11)(p15;p15) can produce more than one *NUP98/HOXA* fusion [16]. We investigated *MSI2* regulation in the first case of Ph-positive CML in blast crisis with t(7;11) and *NUP98/HOXA13* fusion gene.

## Case presentation

### Patient

A 39 year-old man was referred to our department because of leukocytosis, mild anemia, and thrombocytopenia (WBC 180000/mmc, Hb 11.8 g/dl, PLT 130000/mmc) associated with splenomegaly. Chronic Myeloid Leukemia in Blast Crisis was diagnosed on peripheral blood and bone marrow smears. Karyotype was 46,XY,t(9;22)(q34;q11)[6]/46,XY,t(9;22)(q34;q11),t(7;11)(p15;p15) [9]. Hydroxyurea therapy followed by Dasatinib 140 mg/day (40 days), were unsuccessful. The patient did not respond to high-dose ARA-C (3 g/m^2^/day, on days 1-3-5-7) and Daunorubicin (50 mg/m^2^/day on days 1-3-5). Rescue chemotherapy with Clofarabine (40 mg/m^2^/day on days 1–5) and Gemtuzumab-Ozogamicin (3 mg/m^2^/day on day 6) induced prolonged aplasia. Four months after diagnosis the patient underwent unsuccessful bone marrow transplantation from a HLA haploidentical brother.

### Fluorescence In Situ Hybridization (FISH)

**Figure 1:**
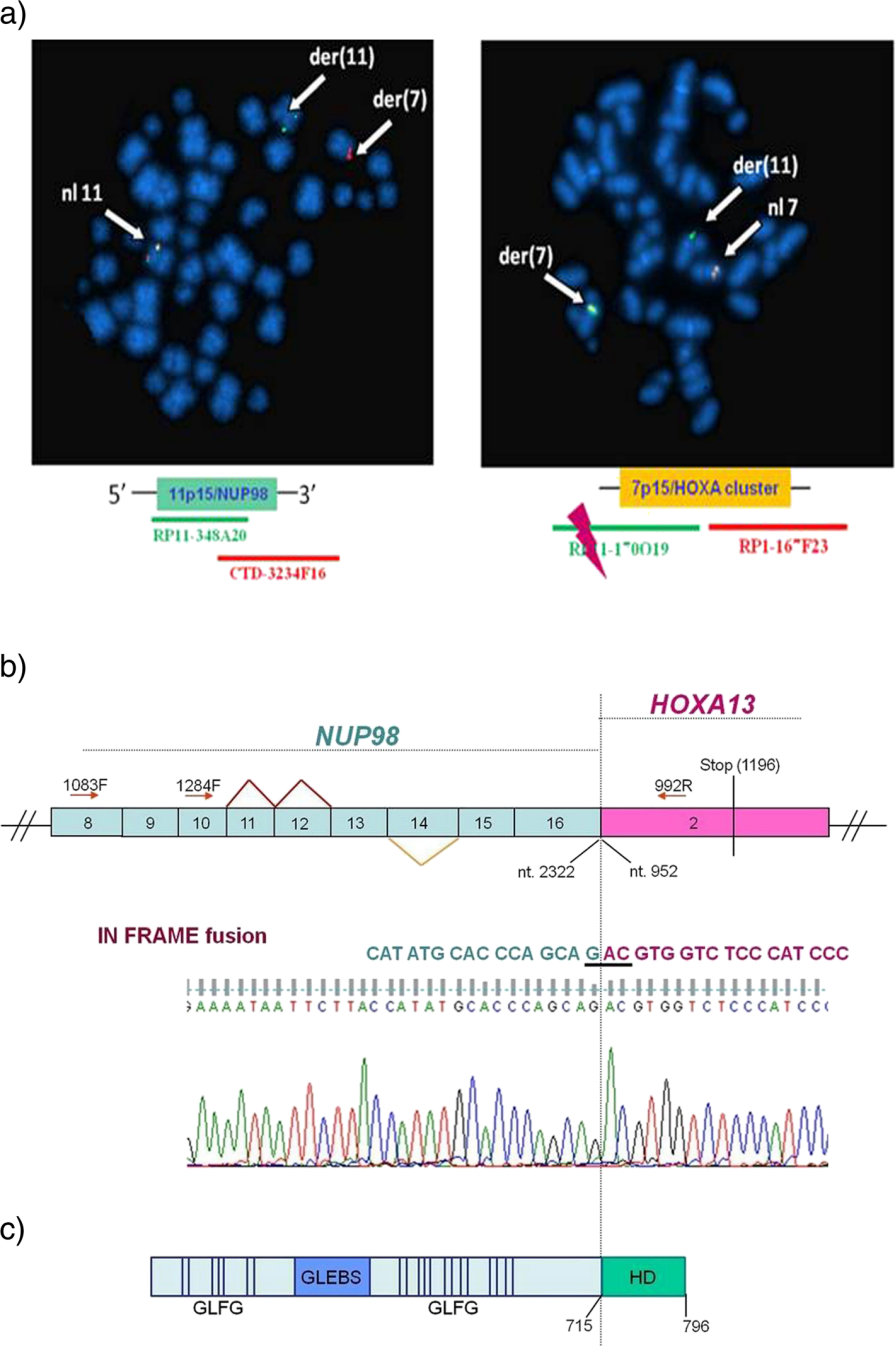
**Molecular and cytogenetic studies. a)** Metaphase FISH showed a rearrangement between *NUP98* gene at 11p15 and the *HOXA* cluster at 7p15. **b)** Breakpoint falls between *NUP98* exon 16 and *HOXA13* exon 2. Red triangles indicate in-frame splicing variants; yellow triangle an out-of-frame variant. Sequence analysis confirmed the transcript*.***c)** The fusion protein with NUP98 GLFG repeats and the HOXA13 homeodomain (HD).

The t(7;11) at diagnosis was investigated by FISH with RP11-348A20 (green) and CTD-3234 F16 (red) for *NUP98* gene, RP1-170O19 (centromeric, green) and RP1-167 F23 (telomeric, red) for the *HOXA* cluster at 7p15. *MSI2* gene (17q22) was studied with genomic clones RP11-166P13, RP11-226 M10, RP11-118E18 and RP11-13H16, oriented from centromere to telomere. Analysis was performed with fluorescence microscopy (Provis, Olympus, Italy) equipped with a cooled CCD camera (Sensys-Photometrics, Tucson AZ, USA) and a SmartCapture software (Vysis, Stuttgart, Germany). Metaphase FISH (Figure 1a) showed a *NUP98/HOXA* gene rearrangement and Interphase FISH (data not shown) identified the fusion in 57% of nuclei.

### Breakpoint cloning

Total RNA was extracted by Trizol reagent (Invitrogen, Carlsbad, CA, USA) and retrotranscribed with Thermoscript (Invitrogen) according to the manufacturer’s protocol. Nested or semi-nested-PCRs were performed to identify the involved *HOXA* partner gene [18]. In testing for the three *HOXA* genes primers were:

1. *NUP98_1083F 5’-ggtaataccagcaccataggacag-3’* and *HOXA9_1036R 5’-tgtggcctgaggtttagagc-3’* or *HOXA9_736R 5’-cagttccagggtctggtgtt-3’* for the first amplification round; *NUP98_1252F 5’-cttactacatttggaagcac-3’* and *HOXA9_736R* or *HOXA9_708R 5’-gggcaccgctttttccgagt-3’* for the second (*NUP98*: NM_00139131.3, *HOXA9*: NM_001164603.1).

2. *NUP98_1083F* and *HOXA11_837R 5’-ctctcggatctggtacttggt-3’* for the first amplification round and *NUP98_1252F* or *HOXA11_837R* for the semi-nested PCR (*HOXA11*: NM_ 005523.5).

3. *NUP98_1083F* and *HOXA13_992R 5’-cctcctataggagctggcat-3’* for the first amplification round and *NUP98_1252F* or *NUP98_1400F 5’-acctgggactcttggaactg-3’* and *HOXA13_992R* for semi-nested PCRs (*HOXA13*: NM_000522.4).

PCR products were sub-cloned into the pGEM-Teasy vector (Promega, Madison, WI) and sequenced by Sanger’s method (ABI 3500 Genetic Analyzer, Applyed Biosystems, Foster City, CA). Molecular analysis revealed an in-frame fusion transcript. *NUP98/HOXA13* had 3885 bp and a breakpoint between exon 16 (nt 2322) of *NUP98* (NM_00139131.3) and exon 2 (nt 952) of *HOXA13* gene (NM_000522.4). Three splicing variants had the same breakpoint but were lacking respectively *NUP98* exon 11, 12 or 14 (which was out of frame) (Figure 1b and c).

### Quantitative PCR

**Figure 2:**
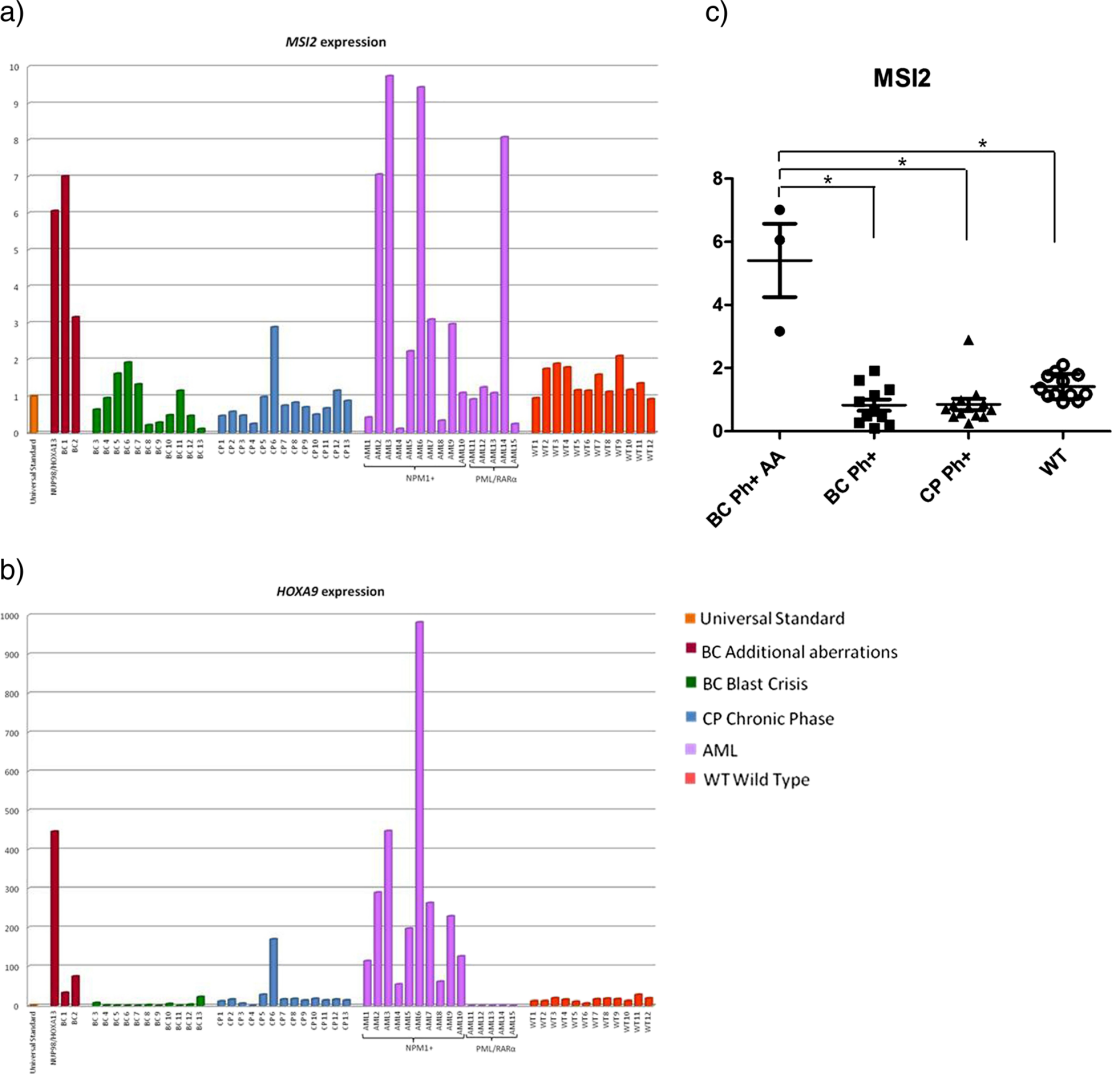
**Expression analysis. a)***MSI2* and **b)***HOXA9* are over-expressed in the present patient with *NUP98/HOXA13*. BC1 and BC2: two other cases of Ph + blast crisis CML with additional karyotypic aberrations over-expressing *MSI2* but not *HOXA9*. Expression values were referred to the average of two references. Fluorescence data were analyzed with the Second Derivative Maximum method; gene expression was expressed as Cp (Crossing point) values. **c)** Significance for *MSI2* expression was tested by Mann–Whitney test (*p < 0.05/3); values are expressed as means +/− SD; AA. Additional aberrations.

Since in silico analysis showed HOXA13 and HOXA9 homeodomains were very similar (75,4% of similarity; 57,9% of identity, score: 274; analysis performed with EMBOSS Matcher program 6.6.0 http://www.ebi.ac.uk/Tools/psa/emboss_matcher/, matrix: BLOSUM80, gap penality: 14, extended penality: 4), we hypothesized NUP98/HOXA13 could bind *MSI2* promoter and tested whether *HOXA9* was involved in the present patient*.* qRT-PCR (LightCycler480, Roche Diagnostics, Germany) was performed using TaqMan assay probes (Applied Biosystems, Foster City, CA) Hs00292670_m for *MSI2* gene, Hs00365956_m1 for *HOXA9* and Hs00426284_m1 for *HOXA13*. Our CML in blast crisis was compared with 41 cases of diverse haematological malignancies (13 blast crisis CML, 13 chronic phase CML, 10 *NPM1c+* AML which over-express *MSI2*[14,20] and 5 Acute Promyelocytic Leukaemia) as well as 12 cases of non-malignant diseases (wt). Reaction mixtures, of 25 μl each, contained 12.5 μl of TaqMan Universal PCR Master Mix (Applied Biosystems), 1.25 μl of the TaqMan assay probe and 5 μl of cDNA (1/10 of RT product). Protocol consisted of 2 minutes at 50°C for activation of AmpliTaq Gold and 10 minutes at 95°C for DNA denaturation. Amplification was performed with 45 cycles of 15 s at 95°C and 1 min at 61°C. All samples were tested in triplicate. Amplification of the sequence of interest was normalized to the average of two endogenous reference controls, *GUSB* (Hs00939627_m1) and *B2M* (Hs00984230_m1) [21], and compared to the expression of a Universal Human Reference RNA (Stratagene, Cedar Creek, TX, USA). Fluorescence data were analyzed with the software version 1.5 and Second Derivative Maximum method; gene expression was expressed as Cp (Crossing point) values. Statistic significance for *MSI2* expression was tested by Mann–Whitney test (*p < 0.05/3). In the present case both *MSI2* and *HOXA9* were over-expressed (Figure 2a, b and c). Notably this over-expression emerged also using the two reference genes singularly [see Additional file 1]. No wild-type *HOXA13* was found (data not shown). *MSI2,* but not *HOXA9*, was significantly over-expressed also in two other cases of Ph + blast crisis CML both harbouring additional cytogenetic aberrations (BC1 and BC2 in Figure 2a and b), with the following karyotypes: 46,XY,t(9;22)(q34;q11) [8]/49-52,XY,idem,+8,+t(9;22)(q34;q11),+13,+15,+21[cp6] (patient BC1), and 46XY,t(3;7)(p21;q32),t(9;22)(q34;q11)[15/15]) (patient BC2). Notably in these cases FISH excluded involvement of *NUP98*, the *HOXA* cluster and *MSI2* gene.

### Chromatin Immunoprecipitation (ChIP)

**Figure 3:**
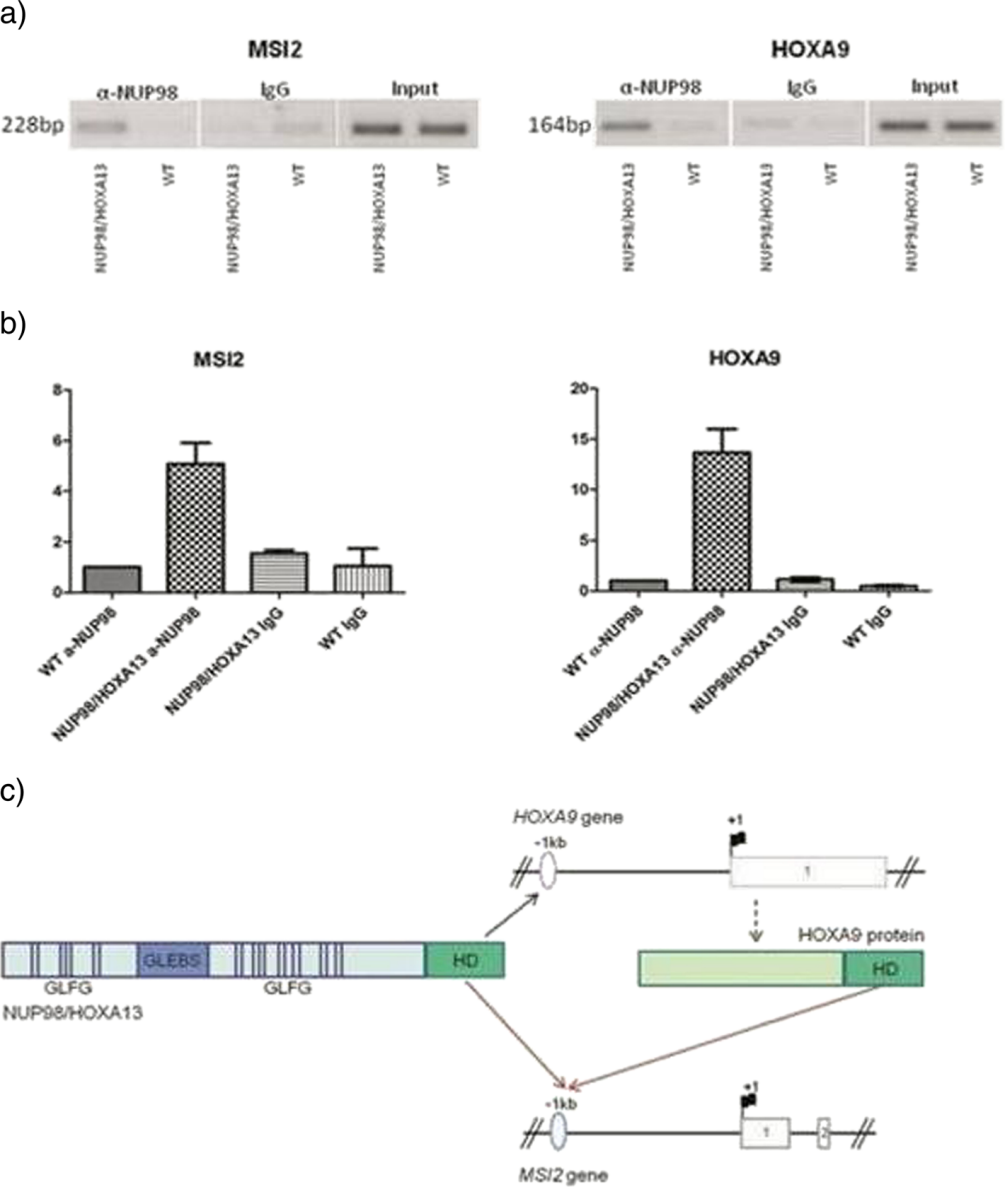
**Chromatin Immunoprecipitation.** NUP98/HOXA13 binds both *MSI2* and *HOXA9* promoters. ChIP was performed on both *NUP98/HOXA13* sample and a non-malignant disease sample (wt). 1,5 μg of rat IgG (Millipore Normal Rat IgG Polyclonal Antibody) and No Antibody (not shown) were used as negative controls. **a)** Semi-quantitative PCR showed an enrichment in NUP98/HOXA13 sample compared to controls. **b)** qPCR confirmed this result; data are presented as fold increase relative to the control sample (wt) based on the formula 2^−ΔΔ*C*p^[23]. One out of three (for *MSI2*) or two (for *HOXA9*) ChIP experiments is shown. The results shown are the mean ± S.E.M. (error bars) of two independent qPCR experiments. **c)** NUP98/HOXA13 binds both *MSI2* and *HOXA9* promoters. HOXA9 binds *MSI2* promoter. Protein structure: homeodomain (HD). Gene structure: exons (numbered boxes), transcription start site (TSS; +1), direction of transcription (flag), putative HOX binding element 1 kb upstream of TSS (oval).

Human *MSI2* and *HOXA9* genomic promoters were deduced using information provided by Web Promoter Scan Service [22]. ChIP assays (EZ-ChIP, Millipore-Upstate, MA, USA) were performed on 2x10^7^ cryopreserved bone marrow cells after cross-linking (1% formaldehyde), lysis (SDS lysis buffer plus protease inhibitors) and sonication. 2×10^6^ cells were pre-cleared and 1% kept as “input”. Anti-NUP98 [2H10] (ab50610 Abcam) was used to recover protein-DNA complexes. 3 μl of immunoprecipitated DNA were amplified by semi-quantitative PCR with primers for the hypothetical HOXA13 binding sites: MSI2_-842 F (5’-GTGTTTGTGCAGGAGGGTCT-3’)/MSI2_-615R (5’-CCCTCTCTAGTTCGCCCTCT-3’) and HOXA9_-1071 F (5’-TAGCAAAGGCGAATTTAAGGG-3’)/HOXA9_-907R (5’- AGTCAAATTCAACGCAGGATC-3’). The exponential amplification conditions were controlled to obtain data in a linear range of amplification. Data were confirmed by qPCR using Brilliant II SYBR Green qPCR Master Mix (Agilent Technologies, USA) and LightCycler480 (Roche Diagnostics, Germany). The crossing point (Cp) determined the quantity of immunoprecipitated DNA [23]. ChIP confirmed the binding of NUP98/HOXA13, but not of wild-type NUP98, to both *MSI2* and *HOXA9* promoters in the present patient (Figure 3a and b).

## Conclusions

This study was important to enrich our knowledge on molecular events underlying the t(7;11)(p15;p15) in the blastic crisis of Ph + CML. For the first time *NUP98/HOXA13* has been identified in CML, although it was reported in one case of AML [17] and one case of MDS [16]. The pathogenetic role of *NUP98/HOXA13* rearrangement in the evolution in this case is emphasized by the absence of transcript isoforms from alternative splicing, such as *NUP98/HOXA11* and all *NUP98/HOXA9,* as previously reported [17]. As we excluded the presence of the wild type *HOXA13* transcript, *MSI2* and *HOXA9* were over-expressed in malignant cells (Figure 2a, b and c) as a consequence of NUP98/HOXA13 fusion. Notably, HOXA13, fused to NUP98*,* up-regulated *MSI2* both directly by binding to its promoter and indirectly by binding to the *HOXA9* promoter, thus inducing a synergistic effect between the two HOXA proteins (Figure 3c).

Over-expression of *MSI2,* but not *HOXA9*, emerged in two other cases of Ph + blast crisis (BC1 and BC2, Figure 2a and b) with additional cytogenetic rearrangements suggesting that, in addition to HOXA9 and HOXA13, alternative mechanisms may deregulate *MSI2* in the presence of clonal evolution and acute phase progression of Ph + CML.

In conclusion this study provided new insights on the molecular heterogeneity of t(7;11)(p15;p15) in the blastic crisis of Ph + CML. Our report suggests that HOXA13 can bind to the promoter of *MSI2* and may contribute to its activation in a patient that harbors NUP98/HOXA13 fusion.

## Consent

Written informed consent was obtained from the patient for publication of this Case report and any accompanying images. A copy of the written consent is available for review by the Editor of this journal.

## Ethics statement

Ethical approval has been obtained for the protocol *“In-depth genomic characterization of leukemia to provide new tools for personalized diagnosis and disease monitoring” (*AIRC 2011–2014) from the University Bioethics Committee of the University of Perugia (Prot. 1.X.2011).

## Abbreviations

MSI2: Musashi2; CML: Chronic myeloid leukemia; AML: Acute myeloid leukemia; B-ALL: B-cell acute lymphoblastic leukemia; BC: Blast crisis; HSC: Hematopoietic stem cell; FISH: Fluorescence in situ hybridization; RT-PCR: Reverse transcription polymerase chain reaction; ChIP: Chromatin immunoprecipitation.

## Competing interests

The authors declare that they have no competing interests.

## Authors’ contributions

DDG designed and performed the research, analyzed the data and wrote the paper, VP performed FISH experiments, GB contributed to data analysis, VC and AV contributed to ChIP experiments and analysis, CM supervised the study and reviewed the manuscript. All authors approved the final manuscript.

## Supplementary Material

Additional file 1 ***MSI2* and *HOXA9 *** **expression in the *NUP98/HOXA13* CML patient and wild type (WT) samples referred to two references genes (a) *GUSB*, (b) *B2M*) singularly.** Click here for file
