# Supplementary material for: Blast crisis Ph+ chronic myeloid leukemia with NUP98/HOXA13 up-regulating MSI2
Source: Mol Cytogenet. 2014 Jun 20;7:42. doi: 10.1186/1755-8166-7-42 (PMC4071805; doi:10.1186/1755-8166-7-42)
Supplement: Additional file 1 — MSI2 and HOXA9 expression in the NUP98/HOXA13 CML patient and wild type (WT) samples referred to two references genes (a) GUSB , (b) B2M ) singularly. [file 1755-8166-7-42-S1.pdf]

Additional file 1

Manuscript ID 1224359181272431

Blast crisis Ph+ Chronic Myeloid Leukemia with *NUP98/HOXA13* up-regulating *MSI2.*

Journal: Molecular Cytogemetics

Danika Di Giacomo, PhD, Valentina Pierini, M.Sc., Gianluca Barba, PhD, Veronica Ceccarelli, PhD, Alba Vecchini, PhD, Cristina Mecucci, MD, PhD.

Corresponding author: Cristina Mecucci MD, Hematology and Bone Marrow Transplantation Unit, University of Perugia, Perugia, Italy.

e-mail address: cristina.mecucci@unipg.it

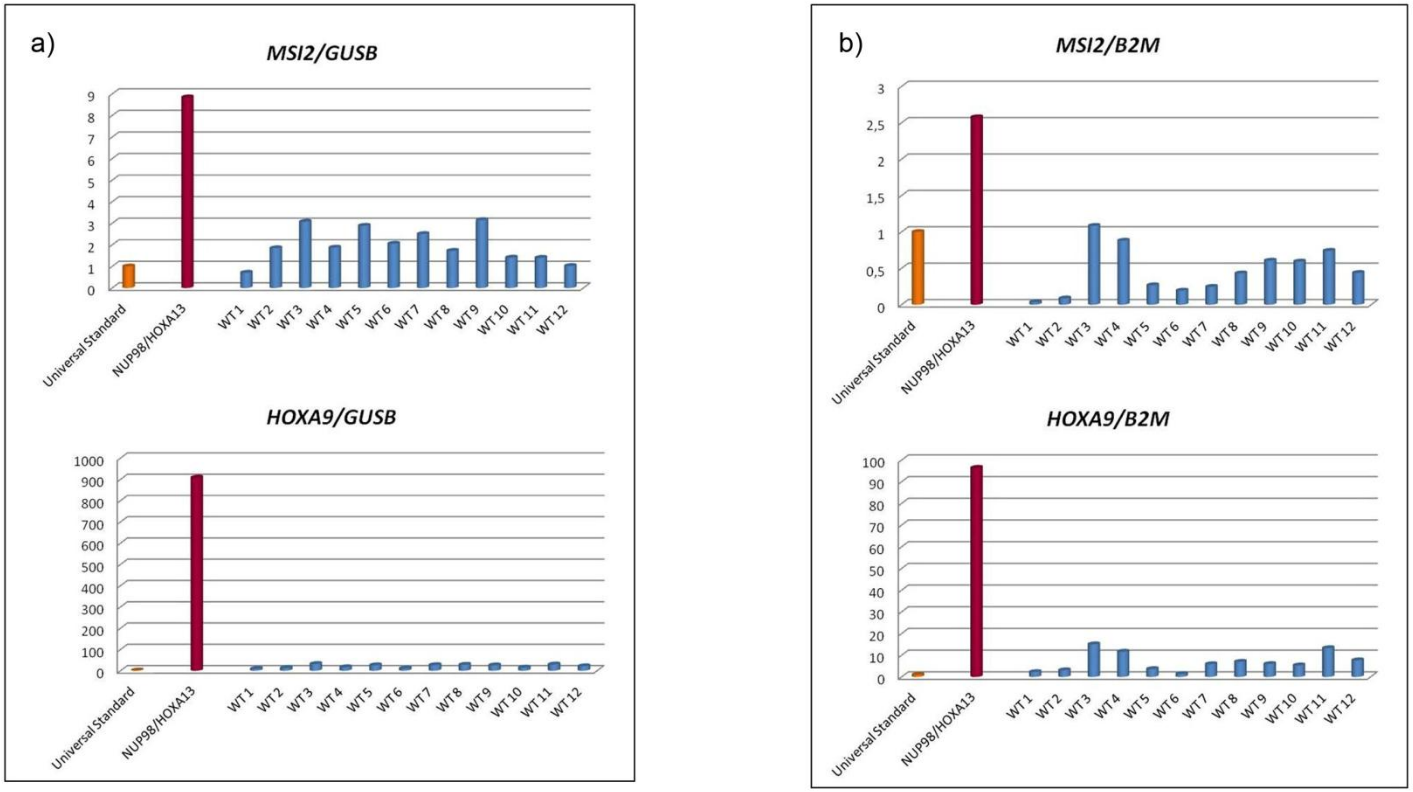

Additional file 1: *MSI2* and *HOXA9* expression in the *NUP98/HOXA13* CML patient and wild type (WT) samples referred to two references genes (a) *GUSB*, b) *B2M*) singularly.
